# Supplementary material for: Different stabilizing mechanisms but a common task-level aim in standing and walking
Source: PLoS One. 2026 Oct 9;21(10):e0355849. doi: 10.1371/journal.pone.0355849 (PMC13652548; doi:10.1371/journal.pone.0355849)
Supplement: Supplementary file 4 [file pone.0355849.s005.docx]

Dear editor,

We would like to thank you and the reviewers for the time and effort invested in reviewing our manuscript and for providing many valuable comments and suggestions. We have carefully revised the manuscript in response to the feedback. In particular, based on the reviewers’ comments, we now refer to our model as a stabilization model rather than a feedback model, have rephrased the introduction, and have revised the description of the aim of the study from investigating feedback control to focusing on the overall stabilizing mechanisms. A detailed point-by-point response to the reviewers’ comments is provided below, and we have also uploaded a revised manuscript with changes highlighted.

Response to reviewers’ comments

**Reviewer #1**

The authors present a study comparing the mechanisms underlying balance during standing and walking, addressing an important question for which substantial gaps in understanding remain. One likely reason for these gaps is that feedback control during walking is inherently difficult to assess, both experimentally and conceptually, due to the closed-loop nature of the system.
While the experiment is carefully conducted and the manuscript is clearly written, I see a major conceptual limitation in the overall study design. Specifically, I do not see how the reported results can be used to answer the stated research question regarding feedback mechanisms. The issue is not one of data quality or analysis, but of methodological suitability.

Major concerns:
1. The study aims to investigate a feedback (control) mechanism, yet the chosen experimental approach does not allow such a mechanism to be identified. In particular, the approach is ill-posed for inferring feedback dynamics in a closed-loop system, as it does not provide a means to disentangle plant dynamics from feedback control. As a result, the underlying control mechanism cannot be uniquely inferred from the observed behavior.

Thank you for this comment. We acknowledge that we cannot interpret what we estimated in this study as the sole result of feedback control. Based on our previous study [1], the model actually estimates the lumped effects of both intrinsic mechanisms and feedback control, i.e., we estimated all mechanisms contributing to stabilization. We realize that we had not made this limitation sufficiently clear in the original version of our manuscript. We used the term feedback rather loosely to encompass neuromuscular feedback and intrinsic stabilizing mechanisms. We now realize that this was confusing. Therefore, to avoid such confusion, we have replaced the term ‘feedback control’ by ‘stabilizing mechanisms’, and have revised our manuscript accordingly. We would like to note that the characterization of the lumped effect of intrinsic dynamics is functionally important, i.e. the results of all stabilizing mechanisms combined determines performance and robustness. At the same our approach does not allow differentiation between intrinsic stabilizing mechanical properties and neurally mediated feedback.

In the abstract, we rephased the purpose as:

Intrinsic mechanisms and feedback control work together to stabilize the body in upright tasks, such as standing and walking. Yet if, and how lumped parameters characterizing stabilization change across tasks is unclear. To answer this question, we applied a stabilization model, relating delayed center of mass position and velocity information to current ground reaction forces (corrected for effects of gravity) in unperturbed standing and walking, to estimate and compare the stabilization between tasks. (lines 19-25)

In the introduction, we mentioned:

Intrinsic mechanisms provide immediate resistance to perturbations based on segmental inertia and passive tissue proprieties (i.e., stiffness and damping). In contrast, feedback control is adaptive and generates corrective responses selectively based on delayed sensory information [2-6]. While identifying these two stabilizing mechanisms separately is methodologically challenging [7], their combined effects determine the performance and robustness of these motor tasks[5, 8]. While studying intrinsic mechanisms and feedback control separately provides insight into the contribution of both mechanisms, characterizing their lumped effects offers an overarching and functional view of how stability is controlled [9]. (lines 50-59)

Since all corrective actions will ultimately be reflected by the ground reaction forces [10, 11], the lumped stabilizing mechanisms can be represented by the relationship between current ground reaction forces and delayed CoM deviations. Based on this, van Dieen et al. [12] proposed a model to assess stabilizing control in unperturbed walking by fitting a linear relationship between the delayed center of mass kinematics and horizontal ground reaction forces. More recently, we refined this model by using the CoM position (PCoM) and velocity (VCoM) information as separate predictors instead of combined into a single predictor, which enabled us to estimate the position- and velocity-related contributions. We now have a framework to evaluate and compare overall stabilization between standing and walking without external perturbations or physiological measurements. The method can provide a better understanding of how the control system regulates global stabilization based on preceding CoM kinematics, and how the parameters characterizing stabilization change across tasks. (lines 86-99)

To address the above questions, we applied a model to quantify stabilization (‘stabilization model’) to unperturbed walking and typical standing postures (normal standing, unipedal, and step postures). (lines 113-116)

2. In this context, it is also important to note that the manuscript makes almost no reference to the substantial body of literature on feedback dynamics in human balance control, primarily in standing but also in walking. This literature explicitly discusses the identifiability limitations of closed-loop balance control and the need for appropriate perturbations or excitation. Several influential studies that are directly relevant to the present work are listed below and should be considered when interpreting feedback in balance control.

We apologize that we did not provide a clear framework when talking about the feedback control in the original manuscript. Thank you for providing the relevant literature, we have carefully reviewed the suggested references and have incorporated some into the manuscript at the appropriate places.

3. More specifically, I see two conceptual problems with the proposed approach.

First, the formulation of the feedback is incorrect. The model equations (eq. 1 in line 182 and eq. 2) do not represent the dynamics of a feedback mechanism. Instead, they describe an open-loop mapping from body kinematics to the force generated by the control mechanism. However, the corrective forces Fcorr also affect PCoM and VCoM, which is not accounted for in the formulation. This self-referential nature—where the sensory input (PCoM,VCoM) affects Fcorr, and Fcorr in turn affects the sensory input—is the core defining feature of feedback control. Ignoring this interdependence breaks the closed-loop description of the system. This mistake leads to biased parameter estimates and, more importantly, to an incorrect representation of the system dynamics.

We agree with your concern. As mentioned in the reply to your first comment, what we estimated should not be interpreted as pure feedback control but as the combined effect of all stabilizing mechanisms.

Interpreted as the feedback control, the estimated values would indeed be biased. Based on our simulation results and tests using estimated human control parameters [1], we can say for bipedal standing that while the estimated values were mainly determined by the actual feedback parameters, increasing intrinsic stabilization introduced a positive bias in the estimated gains and a negative bias in the estimated effective delay.

To address this conceptual issue, we have revised the manuscript to avoid referring to what we estimated as the feedback control, but describe parameters as reflecting the overall effect of all stabilizing mechanisms effective in standing and walking. Please see the answer to your first comment.

4. Second, the experimental approach is limited in its ability to identify feedback (control) mechanisms. Fundamental results from control theory and closed-loop system identification indicate that external perturbations or persistently exciting inputs are generally required to disentangle plant dynamics from feedback control. In the absence of such perturbations, the identification problem becomes ill-conditioned and does not allow unique inference of the underlying control structure. A technical discussion of this issue in the balance control literature is provided by van der Kooij et al. (2005), with similar arguments found in standard system identification textbooks (e.g. Pintelon and Schoukens 2004).
To put this argument in simple terms: sensory systems, neural processing, and the generation of torque all introduce noise, i.e. random fluctuations in the signals of the control system. The properties of this noise (spectral color, magnitude, etc.) are unknown. At the same time, the dynamics of the control system—its structure and parameters—are also unknown. Spontaneous sway behavior during unperturbed stance therefore reflects the combined effect of unknown noise properties and unknown feedback dynamics. Both interact and together give rise to the observed behavior.
When comparing two conditions with different sway patterns, such differences may result from changes in noise properties, changes in feedback dynamics, or a combination of both. In the present experiments, a comparison between walking and standing would require the assumption that internal noise characteristics are identical in both conditions. This assumption is unlikely to hold, as during walking motor noise, and at a minimum also the noise associated with proprioceptive reference to the support surface, differ from those in standing balance. Consequently, it is not possible to determine whether observed differences arise from changes in the control mechanism, changes in noise properties, both, or neither.
The only way to resolve this ambiguity is to apply controlled perturbations and to separate the response to the perturbation from spontaneous sway components. The relationship between perturbation and sway response then characterizes the dynamics of the control system and can be used to identify candidate control mechanisms. This approach is well established in the balance control literature (see Peterka 2002 for a milestone contribution; see also Reimann et al. 2018 and Rafiee & Kiemel 2020 for applications to walking balance).
In summary, feedback control of balance cannot be identified from pure observation when noise sources have unknown characteristics, which constitutes a fundamental limitation when studying closed-loop systems. For the present study, this implies that the proposed experimental approach does not allow the feedback dynamics to be inferred from the collected data.

Thank you for the detailed comments regarding the identification of feedback control. We agree that it is impossible to distinguish the plant dynamics, intrinsic mechanisms and feedback control without applying external perturbations. It is also the main limitation of our model.

We have to admit that we failed to accurately describe what our model really estimates and this led to a misunderstanding on the interpretation of the results. To solve this problem, we have now made it clear throughout the paper that the estimated values should be interpreted as describing the lumped effect of all stabilizing mechanisms in standing and walking, which includes both intrinsic mechanisms and feedback control.

Peterka, Robert J. „Sensorimotor integration in human postural control.“ Journal of neurophysiology 88, Nr. 3 (2002): 1097–118.

Kooij, Herman van der, Edwin van Asseldonk, und Frans C T van der Helm. „Comparison of different methods to identify and quantify balance control.“ Journal of neuroscience methods 145, Nr. 1–2 (2005): 175–203. <https://doi.org/10.1016/j.jneumeth.2005.01.003>.

Peterka, Robert J. „Simplifying the complexities of maintaining balance.“ IEEE engineering in medicine and biology magazine : the quarterly magazine of the Engineering in Medicine & Biology Society 22, Nr. 2 (2003): 63–68.

Mergner, T, C Maurer, und R J Peterka. „A multisensory posture control model of human upright stance.“ Progress in brain research 142, Nr. 1 (2003): 189–201. <https://doi.org/10.1016/S0079-6123(03)42014-1>.

Pasma, J. H., D. Engelhart, A. C. Schouten, H. van der Kooij, A. B. Maier, und C. G.M. Meskers. „Impaired standing balance: The clinical need for closing the loop“. Neuroscience 267 (2014): 157–65. <https://doi.org/10.1016/j.neuroscience.2014.02.030>.

Maurer, C, und R J Peterka. „A new interpretation of spontaneous sway measures based on a simple model of human postural control.“ Journal of neurophysiology 93, Nr. 1 (2005): 189–200. <https://doi.org/10.1152/jn.00221.2004>.

Reimann, H., Fettrow, T., Thompson, E. D., & Jeka, J. J. (2018). Neural control of balance during walking. Frontiers in physiology, 9, 1271.

Rafiee, S., & Kiemel, T. (2020). Multiple strategies to correct errors in foot placement and control speed in human walking. Experimental brain research, 238(12), 2947-2963.

Pintelon, Rik, und Johan Schoukens. System identification: a frequency domain approach. John Wiley & Sons, 2012.

**Reviewer #2**
Understanding whether common control principles govern balance during standing and walking has implications for motor control theory, clinical assessment, and rehabilitation. This study applies a unified feedback control model to unperturbed walking and three standing postures: bipedal, unipedal, and step posture. The authors estimate position and velocity feedback gains and effective delays from center of mass kinematics and ground reaction forces in 15 young adults. Model fits are generally high across tasks. Feedback delays are longer in walking than standing. Normalized position gains exceed critical stiffness in most conditions. The ratio of position to velocity gains approximates the inverted pendulum eigenfrequency across tasks. The authors interpret this as evidence of shared control principles between standing and walking.
This is a meaningful and well-motivated question. Applying one observable feedback model to both walking and standing without perturbations is novel and potentially valuable. The modeling approach is clearly described in many sections. However, several methodological and interpretive issues limit confidence in the strength of the central claim. These concerns are addressable without new data, but they do require clearer justification, potentially additional analyses, and more careful framing of conclusions.

Overall Assessment
The authors address a meaningful problem in locomotor control. The approach is promising and the results are potentially informative. However, the strength of the “shared principles” conclusion currently exceeds what the analyses may support. Methodological inconsistencies, model assumptions, and interpretation issues complicate direct comparisons across tasks. Clarifying these issues and refining the framing of the conclusions would substantially strengthen the manuscript and improve confidence in its claims.

>> Major Points

1. Inconsistent Delay Identification Across Tasks
Although the manuscript applies a “unified” feedback model, the delay identification procedure differs between standing (R² maximization; lines 189-190) and walking (most negative weighted sum of gains; lines 200-201). This inconsistency limits the comparability of feedback parameters across tasks and challenges the claim of a unified modeling framework (lines 182-185).

This matters because delay directly influences gain estimates. If delay is optimized using different criteria across tasks, then differences in delay and gain magnitude may reflect optimization strategy rather than physiological differences. Since the goal is to argue for shared control principles using a unified framework, then the fitting approach and parameter identification must be comparable or at least shown not to bias the comparison.

To resolve this, I recommend that the authors:
1. Either apply the same delay selection criterion to both tasks and reanalyze the data
2. Or provide a clear(er) rationale for why different optimization criteria are necessary and include a sensitivity analysis showing that the main findings hold under alternative delay selection rules.

Without this, it remains unclear whether the reported task differences reflect control strategy or fitting procedure.

We would firstly like to acknowledge that we have rephrased the aims of our paper in view of the conceptual unclarity in the original version. We used the term feedback rather loosely to encompass neuromuscular feedback and intrinsic stabilizing mechanisms. We now realize that this was confusing. The estimated values in this study reflect the lumped effects of both intrinsic and feedback mechanisms rather than purely the feedback control. Accordingly, the aim of the study has been rephrased as to investigate the stabilizing mechanisms in standing and walking.

We agree that using different criteria for delay identification might seems inconsistent without providing reasonable explanation. However, we would like to clarify that this difference is necessary regarding the inherently different temporal dynamics between standing and walking.

Standing, is a quasi-static task, its feedback control is time-invariant or has only little variation over time. In this context, the delay was selected based on the largest percentage of variance accounted for, which is a common goodness fit criterion in model fitting and regression analysis. Our sensitivity analysis from a typical subject also shows that the gains are highly stable in standing, variations of ±20ms in the estimated effective delay (220ms) led to a change of less than 1% of the normalized position gain and 10% of the normalized velocity gain. This supports the robustness and physiology plausibility of the delay selection criteria in standing.

In contrast, walking is a phase-dependent task where the stabilizing mechanisms vary across the gait cycle. In this situation, using the maximum R^2^ is problematic because the center of mass signals exhibit relatively low variance during certain phases. Using the maximum R^2^ to determine the delay yielded physiologically implausible delays (<50ms in the ML direction). Therefore, we used the maximum weighted sum of the position and velocity gains to select the effective delay.

We thus argue that applying different criteria to determine effective delay for standing and walking is appropriate. This difference is, however, inconsistent with the word ‘unified’ in the introduction in the original manuscript. Therefore, we have revised the introduction to avoid using the term ‘unified’, and have added detailed explanation in the method section to clarify the rationale for using different delay selection criteria in standing and walking, as follows:

For standing:

The effective delay was selected based on the model fit (maximum R^2), which is a common goodness of fit criterion in model fitting and regression analysis. (lines 221-223).

For walking:

However, the effective delay could not be determined as the delay at which the best model fit was obtained. During walking, the center of mass signal exhibits relatively low variance during certain phases. As a result, the numerically optimal fit in these phases does not reflect stabilization (e.g., the effective delay in the ML direction selected based on the best fit was less than 50ms, which is physiologically implausible). Thus, we selected the time lag that yielded the most negative weighted sum of position and velocity gains averaged over the stance phase as the effective delay. (lines 231-238).

2. Definition and justification of “corrective” forces in walking
The authors remove gravitational components from walking ground reaction forces based on inverted pendulum model assumptions and refers to the remaining signal as “corrective” force (lines 157-160; lines 166-169). My concern is not that this approach is invalid, but that its justification and implications are insufficiently developed for walking (lines 168-169).

Walking, particularly during double support, departs from single inverted pendulum mechanics (lines 168-169). The model's failure to capture walking complexity during double-stance may lead to systematic errors in force decomposition and these errors could propagate to parameter estimates in ways that systematically bias comparisons with standing.

Also, while model fits improved using corrective forces, this improvement does not necessarily validate the decomposition. Because both the preprocessing force decomposition and the feedback model rely on the same pendulum assumptions, there is potential for the preprocessing to shape the data in ways that favor the model fit that sounds circular in the methods (lines 173-175).

I recommend that the authors consider:
> Provide a clearer mathematical description of how corrective forces were computed. Present the equations explicitly and state all assumptions.

> Consider analyzing single stance separately, where pendulum assumptions are more defensible, or justify why inclusion of double stance does not affect conclusions. A sensitivity check might clarify whether decomposition biases parameter estimate.

This issue influences interpretation of walking parameter estimates and therefore the central comparison with standing. More transparent justification would strengthen confidence in the derived walking parameters.

Thank you for the suggestion. To clarify the computation of the corrected ground reaction forces, we have now added a schematic illustration and some description in the revised manuscript:

The removal process was illustrated in Fig 1, with further details can be found in [1].(lines 180-181)

Fig 1. Removal of the gravity component. In this figure, the human body is modelled as a single inverted pendulum (IP). The left plot represents the IP states, with the dashed line indicating the reference upright state, while the solid line shows a representative IP state with a sway angle $\theta$. In this representative state, the IP experiences the gravitational force ($G$) and generates a ground reaction force ($F_{gr}$) in the ML/AP direction. Due to the inclination, the horizontal $F_{gr}$ includes a passive force resulting from gravity in addition to the ground reaction force generated by muscle activity ($F_{corr})$. The right plot further explains how we calculated the corrected ground reaction force $F_{corr}$. Gravity can be decomposed into two components: a centripetal force ($G_{r}$), which equals $G*\cos\theta$, and a tangential force ($G_{\tau}$), which equals $G*\sin\theta$. The effect of $G_{\tau}$ in both horizontal directions ($G_{\tau'}$) can be calculated as $G_{\tau}*\cos\theta$. Finally, $F_{corr}$ is obtained by $F_{corr}=$ $F_{gr}-G_{\tau'}$. (lines 185-196)

Regarding the second concern, we admit that the single inverted pendulum is not a perfect representation of walking, particularly during the double-support phase. However, double-support phase is a relatively short duration during the whole gait cycle. In addition, the model fits were consistently high across single-, double-support, and step posture, suggesting that applying single inverted pendulum model works in walking. Moreover, the ratio of position to velocity gains close to the eigenfrequency support that the body follows an IP trajectory. We realized that this limitation should be much clearer, and we have added additional description in the limitation section.

First, we modeled the human body as a single inverted-pendulum in both standing and walking. Although this simplification provided good model fits in all tasks, it does not fully capture the multi-segmented nature of the human body, particularly not during the double-support phase of walking. This may have introduced errors in force decomposition. However, the fact that the ratio of position to velocity gains was close to eigenfrequency across tasks suggests that the task-level stabilization achieves a pendulum-like trajectory, which suggests that this simplification does not substantially affect the estimation of overall stabilizing mechanisms in the current study. (lines 537-545)

3. Interpretation of the Position-to-velocity Gain Ratio
The authors interpret the finding that the gain ratio approximates √(g/l) as evidence for shared neural control principles (lines 383-388). However, an inverted pendulum like ratio can arise from shared mechanics alone (line 471-472), independent of neural strategy. This is an interesting result but the interpretation should be more cautious.

I recommend that the authors:
> Reframe the interpretation more cautiously. Explicitly distinguish what this finding reveals about neural control versus what it reveals about whole-body mechanics.
> Consider whether deviations from √(g/l) might be more informative than the approximate match. Deviations from √(g/l) across tasks might reveal how the nervous system adapts control parameters to task-specific demands, whereas approximate consistency primarily confirms that both tasks involve stabilizing a pendular body. Task-specific deviations could indicate functional specialization of control strategies.

These changes would acknowledge alternative explanations rather than narrowly interpreting it as direct evidence of shared neural strategy.

As mentioned in the reply to your first comment, our model estimates the combined effect of both intrinsic mechanisms and feedback control, thus the estimated gains should be interpreted as lumped parameters. Under this interpretation, the observation that the ratio of position to velocity gains is close to the eigenfrequency across conditions reflects that with changing intrinsic stiffness and damping, neural control is adjusted such that the overall behavior keeps the whole-body dynamics close to that of an inverted pendulum.

We agree with your point regarding the deviation from the eigenfrequency, this may provide additional insight into task-specific stabilization characteristics. Following this suggestion, we have further discussed it in the revised manuscript.

The fact that the ratio of lumped gains remained consistent and close to the eigenfrequency across all tasks, suggests that the control system modulates the stabilizing mechanisms to keep the whole-body dynamics close to that of an inverted pendulum.

The deviations from the eigenfrequency ratio, particularly in standing, may reflect task-dependent stabilizing mechanisms. The larger ratio observed in standing compared to walking suggests prioritization of position control over velocity, which aligns with the goal of a static task: keeping a fixed reference point. (lines 528-535)

4. Influence of Intrinsic Stiffness on Delay and Gain Estimates
The authors acknowledge that intrinsic mechanical stiffness bias feedback parameter estimates, particularly the delay parameter. This is mentioned as a limitation but deserves more serious treatment given its potential to confound all major comparisons.

Task differences alter co-contraction levels and intrinsic stiffness (lines 432-442). Changes in intrinsic stiffness can: shorten delays, alter effective gain estimates, and change system dynamics independently of neural feedback. If intrinsic stiffness varies systematically across tasks, then some reported differences in delay and gain magnitude may reflect mechanical changes rather than changes in feedback control.

I recommend that the authors consider:
> Clarify which conclusions are robust to this confound and which should be interpreted cautiously.
> Consider whether intrinsic stiffness could be explicitly incorporated into a model, or explicitly justify why it is treated as a limitation.

Without this clarification, confidence in interpretating task differences remains limited.

Thank you for the comments. In the revised manuscript, we have clarified that our model estimates overall stabilizing mechanisms, rather than estimating feedback alone. From this perspective, any differences caused by intrinsic stiffness does not lead to a bias but behave as part of the overall stabilization. Accordingly, the previously mentioned bias for the estimated feedback values is no longer exist. However, failing to be separating these two mechanisms is a limitation to quantifying their relatively contributions. Moreover, even there are some differences in the intrinsic stiffness and further contribute the lumped values, the main findings that the lumped position to velocity gains remained close to eigenfrequency remains same across tasks, which indicates that the control system modulates the contribution from intrinsic and feedback mechanisms to keep the whole-body dynamics close to that of an inverted pendulum. We have explicitly addressed this in the method, results and limitations of the revised manuscript.

Simulations showed that, for standing, the identified stabilizing parameters are primarily determined by feedback control [1]. While intrinsic stabilization leads to systematic biases: under realistic combination of intrinsic and feedback parameters, increased intrinsic parameters lead to a shorter delay but increased position and velocity gains compared to the input feedback values. These biases should be taken into account when comparing the lumped values across tasks. (lines 273-278)

Among standing postures, unipedal standing and step posture had significantly larger lumped position gains than normal standing, which can partly be attributed to their higher intrinsic stiffness as mentioned above. However, feedback control might also contribute to these differences, and it is impossible to separate the intrinsic and feedback contributions without applying external perturbations [7]. Thus, it remains unclear to what extent feedback mechanisms contribute to the differences in lumped gains. Interestingly, lumped position gains in unipedal standing were approximately twice the per leg values observed in normal standing, despite the smaller base of support and the reduction in number of muscles that can contribute to the corrective forces. This indicates that the control system compensates for the reduced mechanical stability by increasing the overall stabilizing contribution, reflecting a flexible adjustment of the combined stabilizing mechanisms. Similarly, it is reasonable to speculate that the feedback control in step posture might be decreased due to the additional structural stiffness. (lines 500-513)

Furthermore, while the lumped model captures the overall stabilizing effects, separating intrinsic and feedback contributions would provide more detailed information, but this requires perturbation-based studies. (lines 551-553)

5. Treatment of Phase-Dependent Control in Walking
Walking gains vary across stance phase, yet the authors use mean gains to compare walking with time-invariant standing (lines 204-208; lines 268-272). Averaging simplifies the analysis, but it removes temporal structure, an important and distinguishing feature of walking control.

I recommend that the authors:
> Clarify what is meant by “shared principles” throughout the text. Is it similarity in instantaneous control structure, average gain magnitude, or structural relationships such as the gain ratio?
> Address whether phase-dependent control in walking represents a fundamentally different strategy or a modulation of the same underlying control strategy.

This point would strengthen conceptual clarity.

The stabilization gains in walking indeed varied across the gait cycle, while stabilization in standing tasks is non-cyclic and time-invariant. Although it is possible to compare stabilization gains in standing with walking gains at each phase, such comparison does not provide a straightforward representation of the overall stabilization requirement. The mean gains in walking are representative parameters of the overall behavior. Under this task-level interpretation, the similarity of the position-to-velocity ratio suggests their overall stabilization effort, rather than the instantaneous control parameters, produce a similar pendulum-like behavior in maintaining whole-body balance, and the phase-dependent change in walking reflects modulation of the stabilization efforts across the gait cycle.

6. Inverted Pendulum Assumptions and Multisegmented Contributions
The discussion relies heavily on single inverted pendulum interpretations, including critical stiffness and eigenfrequency arguments. While the inverted pendulum model is useful and widely used, both standing and walking involve multi-link coordination. My concern is not that the inverted pendulum model is inappropriate, but that conclusions may extend beyond what the model can justify

I recommend that the authors consider:
> Explaining the specific deviations (multi-link coordination, ankle vs. hip strategies) and their implications for the model. Relate findings to literature evaluating when single pendulum models are adequate and when multi-link behavior dominates.
> Clarifying how much of the observed behavior can reasonably be attributed to inverted pendulum mechanics versus multi‑segment control.

This will prevent overgeneralization and strengthen the credibility of the conclusions.

We agree that standing and walking both involve multi-link coordination and that ankle, hip and other segment strategies contribute to stabilization. The single inverted pendulum is too simple to completely account for the whole-body mechanics in both tasks.

However, the single inverted pendulum assumption is only used to estimate the gravitational component of the total ground reaction forces. It represents the net effects of all segments’ contributions rather than how individual segments contribute.

More importantly, our main conclusion does not rely on the IP assumption. The observed ratio close to the eigenfrequency across tasks suggests that the control system regulates behavior in a way that approximates inverted pendulum dynamics at the task-level. This suggests that the overall stabilizing effects may coordinate the multi-segment efforts to produce the pendulum-like trajectory, which aligns with the idea that angular and linear momentum are regulated in a coordinated manner[13].

In the limitation, we mentioned:

First, we modeled the human body as a single inverted-pendulum in both standing and walking. Although this simplification provided good model fits in all tasks, it does not fully capture the multi-segmented nature of the human body, particularly not during the double-support phase of walking. This may have introduced errors in force decomposition. However, the fact that the ratio of position to velocity gains was close to eigenfrequency across tasks suggests that the task-level stabilization achieves a pendulum-like trajectory, which suggests that this simplification does not substantially affect the estimation of overall stabilizing mechanisms in the current study. (lines 537-545)

7. Task Definitions: Unipedal and Step Posture Clarifications

Unipedal Stance (lines 129-130): The position of the non-stance leg is not clearly described. This detail matters because leg position alters stability demands.

Please specify:
> Was the leg held behind (like initial swing/toe-off), ahead (like terminal swing/pre-heel-strike), or adjacent (like mid-swing)?
> Were hip and knee angles standardized?
> Was this controlled across participants and trials?

The introduction states these postures resemble behaviors during single support in walking. If that is the intent, specify which swing phase posture was modeled and justify the choice.

For unipedal standing, we asked participants to stand on the support leg while keeping the non-support leg in a position in which they could maintain stability. The position of the non-support leg was therefore not standardized in terms of hip and knee angles and different participants used different leg positions. Our aim in this task was not to replicate a specific phase during single-support in walking, but rather to create a single-support posture with a reduced base of support compared to double support. Moreover, various leg configurations naturally occur across the single-support phase.

In the introduction, we added:

To address the above questions, we applied a model to quantify stabilization (‘stabilization model’) to unperturbed walking and typical standing postures (normal standing, unipedal, and step postures). Unipedal standing and step posture were selected as they are commonly observed during walking, while they provide a different base of support (BoS) and different structural stiffness compared with normal standing. Comparing these postures allows us to determine whether a consistent position-to-velocity control ratio exists across different biomechanical contexts. (lines 113-120)

in the method, we added:

2) Unipedal standing: stand on the preferred leg with the other leg in a position in which the participants could maintain stability. (lines 145-146)

Step Posture (lines 132-135): The step posture imposed equal weight distribution between feet. In natural double support, weight distribution is dynamic and asymmetric.

Please justify:
> Why 50 percent weight distribution was selected.

> Why initial or terminal double support, which may better resemble walking mechanics, was not modeled.

We chose the current step configuration, because different participants preferred different weight distributions between two feet. This led to task differences. Therefore, we decided to ask them to adopt a step posture with their center of mass in the middle between the two feet. In our pilot study, we actually applied step postures with body weight on the front leg, rear leg and in the middle, there was no systematic significant differences between them.

In the text, we have added:

This even distribution was selected as our pilot study showed no consistent differences between different weight-distributions. (lines 152-153)

> Whether a single inverted pendulum model is appropriate for bipedal stance with two contact points and a base of support.

Although bipedal stance involves two contact points, at the center-of-mass level the net ground reaction forces from both limbs can be represented as a single resultant force, allowing the system to be approximated as a single inverted pendulum for overall stabilization.

Clarification of whether the step posture is meant to represent double‑support mechanics or simply serve as an additional standing configuration needs to be stated clearly.

The step posture, as well as the unipedal standing, cannot replicate the detailed mechanics of the double support phase in walking, but an additional standing configuration with a base of support that resembles that adopted during certain phases in walking. We have clarified this in the revised version.

To address the above questions, we applied a model to quantify stabilization (‘stabilization model’) to unperturbed walking and typical standing postures (normal standing, unipedal, and step postures). Unipedal standing and step posture were selected as they are commonly observed during walking, while they provide a different base of support (BoS) and different structural stiffness compared with normal standing. Comparing these postures allows us to determine whether a consistent position-to-velocity control ratio exists across different biomechanical contexts. (lines 113-120)

>>Minor Points

Thank you for the following detailed comments on the manuscript. We have made appropriate revisions point-by-point in response to your suggestions.

Introduction
> The first two paragraphs are largely under-cited. Add references on feedback control in standing and walking, and distinct feedback strategies.
> Improve organization and conceptual flow. The jump from standing vs. walking differences to feedback models to XCoM concept could be smoother. Consider reorganizing.
> Introduce and explain XCoM more clearly when first mentioned (lines 69-70). The explanation of XCoM and its predictions comes several paragraphs after it is first mentioned (lines 88-90).

Methods
> Use SI units consistently. Report walking speed as 1.25 m/s and optionally include km/h in parentheses (line 125).

In the revised manuscript, we have modified the units:

For walking, participants walked on the instrumented dual-belt treadmill (Motek-Force-link, Amsterdam, Netherlands) with belt speed set at 1.25 m/s (4.5 km/h) until a steady gait was achieved, followed by an additional 5 min walking trial. (lines 139-142)

> Justify exclusion of the first 6 seconds and last 10 seconds from standing trials (line 161).

Please see text in lines 199-202:

For model fitting, we analyzed experimental data from seconds 6 to 50 of each trial. This interval was selected to exclude initial postural adjustments at the start and increased body sway at the end of the task, particularly in unipedal standing.(lines 181-184)

> Specify how ground reaction forces collected during standing postures. Confirm whether participants stood on the instrumented treadmill used during the walking trials (lines 128-141).

In short, we collected whole-body 3D kinematic data using Optotrak cameras (Northern Digital Inc., Waterloo, Ontario, Canada) and ground reaction forces with force plates embedded in the treadmill (Motek-Force-link, Amsterdam, Netherlands), and processed these data using MATLAB (2024a, The MathWorks, Natick, US). (lines 162-166)

For standing tasks, participants stood on the instrumented dual-belt treadmill and were asked to perform: 1) Normal standing: stand upright with feet together. 2) Unipedal standing: stand on the preferred leg with the other leg in a position in which the participants could maintain stability. (lines 143-146)

Results
> Table 1 reports partial eta squared values but does not interpret them. Provide brief interpretation of effect sizes and discuss practical significance.

Effect size was evaluated using partial eta squared (${\eta^{2}}_{p}$), with values of 0.01, 0.06, and 0.14 considered as small, medium, and large effects, respectively[14] (lines 289-291)

> State what level of explained variance (R2) is considered acceptable for drawing conclusions (lines 248-249).

Similar to our previous study[1], we considered $F_{corr}$ to be sufficiently reconstructed when $R^{2}$ ≥ 0.70. (lines 293-294)

Discussion
> Clarify what is novel if similarities primarily reflect shared whole-body mechanics. Clarify what the study reveals about neural control versus body mechanics.

Please see our reply to your 3rd comment ‘Interpretation of the Position-to-velocity Gain Ratio’.

How do the authors reconcile finding both similarities (gain ratios) and large differences (delays, individual gain magnitudes)? What level of difference would falsify the 'shared principles' hypothesis?

We acknowledge that while effective delays and lumped gains differ substantially across tasks, the ratio between position and velocity gains remains consistent and close to the system’s eigenfrequency. We interpret this as reflecting different stabilizing mechanisms across tasks while with a common task-level goal: making the CoM to behave a pendulum-like trajectory.

The authors should consider reframing the title to reflect both commonalities and task-specific differences to better match the actual findings

We have changed the title to ‘Different stabilizing mechanisms but a common task-level aim in standing and walking’.
The authors should be more explicit about what constitutes a "shared principle" vs. task-specific adaptation. Is it just the gain ratio, or are there other features?
Thanks, you could see the reply to your 3rd comment 3 ‘Interpretation of the Position-to-velocity Gain Ratio’.
>> Recommendation: Minor Revision
The authors address an important problem and use a promising modeling approach. However, several methodological inconsistencies and interpretive issues limit confidence in the central “shared principles” conclusion. These concerns appear addressable through clearer justification, targeted sensitivity analyses, and more careful framing. With these revisions, the manuscript could make a meaningful contribution to understanding feedback control in standing and walking.

Reviewer #3: General comments

This paper addresses an interesting open question in motor control, namely, the adaptability of control schemes between different motor tasks. This paper aims to examine how the parameters in a common feedback control structure compare for standing and walking.

Unfortunately, conceptual flaws in the paper make the argument presented entirely unconvincing. To plausibly demonstrate that a feedback control system is playing a role in a system requires at least two things: a target or set point of some quantity of relevance to the system’s performance, and a demonstration that some form of state feedback is able to drive the system to that target value. The “feedback models” in the authors’ Eqs. (1) and (2) do neither.

This paper considers a point mass walker with its state given by the position and velocity of the center of mass (CoM). What the authors have demonstrated is that, for both standing and walking, the state is linearly correlated to a reasonable degree with the forces generated at the next time. They also identify the time lag that yields the best correlation. But they never show that this supposed control is being used to regulate the actual walking dynamics in any way: for that, one would presumably have to show how the given force is used to create the next CoM state. Indeed, the paper refers to “corrective ground reaction forces” multiple times without ever stating what, exactly, is being corrected. It is merely assumed that the forces they have measured are being used to correct something, but what is not at all clear.

They also find that the correlation parameters obtained in the four cases examined differ and thereby interpret this as “adaptation”. This is at best an hypothesis, and does not indicate a common controller is active in the different situations. The fact that one can find a reasonable linear correlation between the CoM state and force for the different tasks hardly shows that they are using the same controller architecture. Correlation is not causation, but even assuming the CoM state is causing the subsequent force, any mechanism for doing that would cause the two cases to show correlations and, assuming the controller was differentiable, they would both have a linear limit and, hence, could be expected to reasonably satisfy Eqs. (1) or (2). So the fact that one can estimate parameters in all of the cases in no way convincingly demonstrates “adaptation”.

The data collection and analysis methodologies are sound and, as such, the paper has potential to be published in PLOS ONE. However, the interpretations are poorly drawn because of the above-indicated fundamental problems. I cannot, therefore, recommend the paper for publication in its current form. In addition to addressing the specific comments below, the authors need to rewrite the introduction and discussion to more convincingly relate the simple correlation analyses they are doing to the claims about control they are making.

Thank you for this comment. We acknowledge that we cannot interpret what we estimated in this study as the sole result of feedback control. Based on our previous study [1], the model actually estimates the lumped effects of both intrinsic mechanisms and feedback control, i.e., we estimated all mechanisms contributing to stabilization. We realize that we had not made this limitation sufficiently clear in the original version of our manuscript. We used the term feedback rather loosely to encompass neuromuscular feedback and intrinsic stabilizing mechanisms. We now realize that this was confusing. Therefore, to avoid such confusion, we have replaced the term ‘feedback control’ by ‘stabilizing mechanisms’, and have revised our manuscript accordingly. We would like to note that the characterization of the lumped effect of intrinsic dynamics is functionally important, i.e. the results of all stabilizing mechanisms combined determines performance and robustness. At the same our approach does not allow differentiation between intrinsic stabilizing mechanical properties and neurally mediated feedback.

In the abstract, we rephased the purpose as:

Intrinsic mechanisms and feedback control work together to stabilize the body in upright tasks, such as standing and walking. Yet if, and how lumped parameters characterizing stabilization change across tasks is unclear. To answer this question, we applied a stabilization model, relating delayed center of mass position and velocity information to current ground reaction forces (corrected for effects of gravity) in unperturbed standing and walking, to estimate and compare the stabilization between tasks. (lines 19-25)

In the introduction, we mentioned:

Intrinsic mechanisms provide immediate resistance to perturbations based on segmental inertia and passive tissue proprieties (i.e., stiffness and damping). In contrast, feedback control is adaptive and generates corrective responses selectively based on delayed sensory information [2-6]. While identifying these two stabilizing mechanisms separately is methodologically challenging [7], their combined effects determine the performance and robustness of these motor tasks[5, 8]. While studying intrinsic mechanisms and feedback control separately provides insight into the contribution of both mechanisms, characterizing their lumped effects offers an overarching and functional view of how stability is controlled [9]. (lines 50-59)

Since all corrective actions will ultimately be reflected by the ground reaction forces [10, 11], the lumped stabilizing mechanisms can be represented by the relationship between current ground reaction forces and delayed CoM deviations. Based on this, van Dieen et al. [12] proposed a model to assess stabilizing control in unperturbed walking by fitting a linear relationship between the delayed center of mass kinematics and horizontal ground reaction forces. More recently, we refined this model by using the CoM position (PCoM) and velocity (VCoM) information as separate predictors instead of combined into a single predictor, which enabled us to estimate the position- and velocity-related contributions. We now have a framework to evaluate and compare overall stabilization between standing and walking without external perturbations or physiological measurements. The method can provide a better understanding of how the control system regulates global stabilization based on preceding CoM kinematics, and how the parameters characterizing stabilization change across tasks. (lines 86-99)

To address the above questions, we applied a model to quantify stabilization (‘stabilization model’) to unperturbed walking and typical standing postures (normal standing, unipedal, and step postures). (lines 113-116)

Regarding the interpretation of differences between tasks, we have also refined the term ‘adaptation’ to task-dependent stabilizing control with a common task-level goal: making the CoM to behave a pendulum-like trajectory. In particular, while the effective delay and stabilizing gains varied significantly across tasks, the ratio of position to velocity gains remained consistent and close to the system’s eigenfrequency.

We hope these revisions address your concerns and provide a more accurate and appropriately constrained interpretation of our findings.

Specific comments

1. The authors cite only one model of feedback control in walking (the recent paper by Afshcrift et al.). There are many such models. A more representative list and a brief discussion how this study’s definition of control fits in with them will greatly help the manuscript and may help justify your claims about control and adaptation.

Thank you for this comment, we have added more related references when describing the feedback model, mainly in the introduction section.

2. Eq. (1) is a standard AR model with fixed coefficients, whereas Eq. (2) has time (or state) dependent coefficients that are estimated at each phase of a step. The second model is therefore not just an adaptation of the first (or vice versa). To show adaptation, you would need to have exactly the same model in both cases, and show that only its parameters needed to change to obtain comparable results.

As mentioned in the reply to your general comments, we did not estimate feedback control in the present study, but all stabilizing mechanisms. We applied different stabilization model is due to the distinct temporal differences between standing and walking. We also do not suggest that Eq. 2) represents an adaptation of Eq. 1), but both formulations share the same stabilization goal, i.e., stabilization mechanisms correct the center of mass deviations by generating appropriate ground reaction forces. Changes of stabilization gains may indicate how position and velocity are overall controlled.

3. Furthermore, the data you’re fitting in each case is not in the same category: standing gives data that is close to Brownian motion, walking is more like a noisy periodic signal. The time scales over which things are happening in both cases are not remotely the same, so the idea that the same controller is just “adapting” from one case to the next is doubtful.

In the revised manuscript, our purpose has been changed to compare the lumped stabilizing effects between standing and walking. Therefore, there will be no ‘adaption’, but how the stabilization is achieved by coordination of intrinsic and feedback mechanisms.

4. My understanding is that for standing you used 46 seconds of 50 Hz sampled data for the regression, so that’s 2300 data points. Presumably this data appears to be similar to a random walk (in the sense of a random process). In contrast, for walking, you used 5 minutes of walking data, so 300 seconds (and about 300 steps) which, sampled at 50Hz gives 15,000 data points. But you estimate each gain at each of 100 points in a step, so only 300 points are used for each such estimate. This is not only confusing, in the end these regression calculations are not similar to each other so it’s difficult to see how you can compare the two from that point of view. And, again, just because you manage to find reasonably good linear regressions for the different tasks does not imply that the same underlying controller is adapting from one to the other.

Thank you for this comment. As clarified above, our analysis does not aim to identify feedback control, but to estimate the combined effect of all stabilizing mechanisms at the center-of-mass level.

In view of the distinct temporal structure between standing and walking, different modeling approaches have been applied. However, our comparison focuses on the lumped stabilizing mechanisms.

5. Finally, as you point out, standing and walking are quite different in that the latter is rhythmic. Hence, the dynamics observed in each case come from very different physiological processes (e.g. walking involves a CPG, standing does not). So, again, it seems highly doubtful that such different tasks use the same underlying control scheme that is merely “adapted” from one to the other.

We agree that standing and walking involve different physiological processes, and it is unreasonable to assume a same control structure. Since we do not estimate control using the current framework but overall task-level stabilization, we no longer used the ‘adaption’ or similar descriptions.

6. Your study of control only looks at force production. But, as the authors know and have discussed in other works, basic stepping properties (e.g. step width) are known to be very important to maintaining stable walking. Indeed, the very point of Hof’s XCoM and MoS concepts is to look at the relationship between the CoM and the foot position (or, more precisely, the center of pressure location). Why is it that you don’t need to pay any attention to where the feet are placed in this study of control?

The foot placement and CoP modulation are indeed important for balance control during walking. However, all control actions can finally be represented by ground reaction forces. As shown by van Dieen, Bruijn (15), CoP shifts do not directly affect linear CoM momentum, but influence it through the effects on ground reaction forces. Therefore, by focusing on forces, our approach can represent the overall effects of all stabilizing mechanisms. Moreover, since we aimed to compare stabilization between standing and walking, focusing on the foot placement would not be appropriate.

7. It would be good to show representative data. Since you estimated K, B and tau for each phase of walking, it would be good to show what those time series looked like when plotted against phase: are they just noisy, or are they structured in some way?

Thank you for the suggestion. We agree that representative data are useful for understanding whether the phase-dependent estimates of control parameters reflect structured changes across the gait cycle rather than noise. In the manuscript, Fig. 3 showed how the estimated lumped position and velocity gains varied over the gait cycle. In the revised manuscript, we also added representative examples of PCoM, VCoM, and corrected ground reaction forces in the Supplementary Materials as S1 Fig.

**Fig 3. Stabilization models identified in walking with the total (panel A) and corrected ground reaction forces (panel B) as predicted variables.** From left to right: model fit ($R^{2}$), normalized position gains ($\hat{K_{n}}$) and velocity gains ($\hat{B_{n}}$) across the gait cycle. The green and orange lines represent the averaged results (over participants) in ML and AP direction, respectively. The dashed areas indicate the 95% confidence intervals. The horizontal lines in the left panels indicate when the model fit was significant for each individual participant (p<0.05). (lines 338-343)

**S1 Fig. Time series of the PCoM (panel A), VCoM (panel B) and corrected ground reaction forces (panel C) during walking in the AP direction from a typical participant**.

1.	Geng Y, Bruijn SM, van Dieen JH. Feedback control stabilizing the center of mass can be identified in unperturbed, upright standing. Hum Mov Sci. 2026;106:103470. Epub 20260228. doi: 10.1016/j.humov.2026.103470. PubMed PMID: 41764868.

2.	Forbes PA, Chen A, Blouin JS. Sensorimotor control of standing balance. Handb Clin Neurol. 2018;159:61-83. doi: 10.1016/B978-0-444-63916-5.00004-5. PubMed PMID: 30482333.

3.	Shanbhag J, Fleischmann S, Wechsler I, Gassner H, Winkler J, Eskofier BM, et al. A sensorimotor enhanced neuromusculoskeletal model for simulating postural control of upright standing. Front Neurosci. 2024;18:1393749. Epub 20240515. doi: 10.3389/fnins.2024.1393749. PubMed PMID: 38812972; PubMed Central PMCID: PMCPMC11133552.

4.	Bottaro A, Casadio M, Morasso PG, Sanguineti V. Body sway during quiet standing: is it the residual chattering of an intermittent stabilization process? Hum Mov Sci. 2005;24(4):588-615. Epub 20050906. doi: 10.1016/j.humov.2005.07.006. PubMed PMID: 16143414.

5.	Peterka RJ. Sensorimotor integration in human postural control. J Neurophysiol. 2002;88(3):1097-118. Epub 2002/09/03. doi: 10.1152/jn.2002.88.3.1097. PubMed PMID: 12205132.

6.	Asslander L, Peterka RJ. Sensory reweighting dynamics in human postural control. J Neurophysiol. 2014;111(9):1852-64. Epub 20140205. doi: 10.1152/jn.00669.2013. PubMed PMID: 24501263; PubMed Central PMCID: PMCPMC4044370.

7.	van der Kooij H, van Asseldonk E, van der Helm FC. Comparison of different methods to identify and quantify balance control. J Neurosci Methods. 2005;145(1-2):175-203. Epub 20050319. doi: 10.1016/j.jneumeth.2005.01.003. PubMed PMID: 15922036.

8.	Fitzpatrick R, Burke D, Gandevia SC. Loop gain of reflexes controlling human standing measured with the use of postural and vestibular disturbances. J Neurophysiol. 1996;76(6):3994-4008. doi: 10.1152/jn.1996.76.6.3994. PubMed PMID: 8985895.

9.	Reimann H, Fettrow T, Thompson ED, Jeka JJ. Neural Control of Balance During Walking. Front Physiol. 2018;9:1271. Epub 20180913. doi: 10.3389/fphys.2018.01271. PubMed PMID: 30271354; PubMed Central PMCID: PMCPMC6146212.

10.	Shiozawa K, Lee J, Russo M, Sternad D, Hogan N. Frequency-dependent force direction elucidates neural control of balance. J Neuroeng Rehabil. 2021;18(1):145. Epub 20210925. doi: 10.1186/s12984-021-00907-2. PubMed PMID: 34563223; PubMed Central PMCID: PMCPMC8466643.

11.	Sugimoto-Dimitrova R, Shiozawa K, Gruben KG, Hogan N. Frequency-domain patterns in foot-force line-of-action: an emergent property of standing balance control. J Neurophysiol. 2024;132(5):1445-56. Epub 20241003. doi: 10.1152/jn.00084.2024. PubMed PMID: 39361732.

12.	van Dieen JH, Bruijn SM, Afschrift M. Assessment of stabilizing feedback control of walking: A tutorial. J Electromyogr Kinesiol. 2024;78:102915. Epub 20240622. doi: 10.1016/j.jelekin.2024.102915. PubMed PMID: 38936234.

13.	van Dieën JH, Bruijn S, Lemaire KK, Kistemaker DA. Simultaneous stabilizing feedback control of linear and angular momentum in

human walking. 2026. doi: 10.1101/2025.01.09.632072.

14.	Cohen J. Statistical Power Analysis for the Behavioral Sciences. Hillsdale, NJ: Lawrence Erlbaum Associates; 1988.

15.	van Dieen JH, Bruijn SM, Lemaire KK, Kistemaker DA. Simultaneous stabilizing feedback control of linear and angular momentum in human walking. J Biomech. 2026;200:113240. Epub 20260302. doi: 10.1016/j.jbiomech.2026.113240. PubMed PMID: 41791265.
