# Supplementary material for: Different stabilizing mechanisms but a common task-level aim in standing and walking
Source: PLoS One. 2026 Oct 9;21(10):e0355849. doi: 10.1371/journal.pone.0355849 (PMC13652548; doi:10.1371/journal.pone.0355849)
Supplement: Supplementary file 6 [file pone.0355849.s007.docx]

Dear editor and reviewers,

We thank you for your time and effort invested in processing previous versions of our manuscript. After acceptance of the manuscript, we identified an error in the code for processing the data on walking that affected part of the results. Below we provide a detailed explanation of the error, the corrections we have made, and the impact on our results and conclusions.

Specifically, in our analysis, the center of mass (CoM) position should be re-referenced to the leading foot position. Because our experiment was performed on the treadmill, the movement of treadmill belt should be accounted for when calculating the new CoM position. However, this correction was neglected. As a result, the CoM position was calculated incorrectly, which subsequently affected the calculation of the corrected ground reaction forces used in the stabilization model, and therefore the walking results.

In the previous analysis, our results suggested that using corrected ground reaction forces was more appropriate in walking, but this was caused by the larger deviation of CoM due to the lack of correction for treadmill belt movement. After correcting this error, our updated results showed that using the total ground reaction forces was more appropriate than simply removing the gravitational component.

The error affected the several outcomes in walking and statical results, but the main findings were unchanged: while the individual parameters differed significantly, the ratio of position to velocity gains remained close to the body’s eigenfrequency whenever stabilization was pronounced, reflecting a common task-level goal: keeping the CoM on a nominal pendulum-like trajectory.

We have corrected the walking analysis and updated the code on Zenodo. All affected results, figures, tables, and corresponding text have been revised. Both a clean revised manuscript and a version with changes highlighted have been provided.
