# Supplementary material for: Different stabilizing mechanisms but a common task-level aim in standing and walking
Source: PLoS One. 2026 Oct 9;21(10):e0355849. doi: 10.1371/journal.pone.0355849 (PMC13652548; doi:10.1371/journal.pone.0355849)
Supplement: Peer review history

# Peer review: Decision Letter 0

**DOI:** 10.1371/journal.pone.0355849.r001

**Authors:** Anne Martin

24 Feb 2026

Dear Dr. Bruijn,

Thank you for submitting your manuscript to PLOS ONE. After careful consideration, we feel that it has merit but does not fully meet PLOS ONE’s publication criteria as it currently stands. Therefore, we invite you to submit a revised version of the manuscript that addresses the points raised during the review process.

This will require re-framing the paper so that the objectives and interpretation match what the experimental procedure actually allows you to test. In addition to the significant revisions required to re-frame the paper, please also address all other reviewer comments.

Please submit your revised manuscript by Apr 10 2026 11:59PM. If you will need more time than this to complete your revisions, please reply to this message or contact the journal office at plosone@plos.org. When you're ready to submit your revision, log on to https://www.editorialmanager.com/pone/ and select the 'Submissions Needing Revision' folder to locate your manuscript file.

- A letter that responds to each point raised by the academic editor and reviewer(s). You should upload this letter as a separate file labeled 'Response to Reviewers'.
- A marked-up copy of your manuscript that highlights changes made to the original version. You should upload this as a separate file labeled 'Revised Manuscript with Track Changes'.
- An unmarked version of your revised paper without tracked changes. You should upload this as a separate file labeled 'Manuscript'.

If you would like to make changes to your financial disclosure, please include your updated statement in your cover letter. Guidelines for resubmitting your figure files are available below the reviewer comments at the end of this letter.

If applicable, we recommend that you deposit your laboratory protocols in protocols.io to enhance the reproducibility of your results. Protocols.io assigns your protocol its own identifier (DOI) so that it can be cited independently in the future. For instructions see: https://journals.plos.org/plosone/s/submission-guidelines#loc-laboratory-protocols. Additionally, PLOS ONE offers an option for publishing peer-reviewed Lab Protocol articles, which describe protocols hosted on protocols.io. Read more information on sharing protocols at https://plos.org/protocols?utm_medium=editorial-email&utm_source=authorletters&utm_campaign=protocols.

We look forward to receiving your revised manuscript.

Kind regards,

Anne E. Martin

Academic Editor

PLOS One

Journal Requirements:

When submitting your revision, we need you to address these additional requirements.

1. Please ensure that your manuscript meets PLOS ONE's style requirements, including those for file naming. The PLOS ONE style templates can be found at

https://journals.plos.org/plosone/s/file?id=wjVg/PLOSOne_formatting_sample_main_body.pdf and

https://journals.plos.org/plosone/s/file?id=ba62/PLOSOne_formatting_sample_title_authors_affiliations.pdf

2. We note that the grant information you provided in the ‘Funding Information’ and ‘Financial Disclosure’ sections do not match.

When you resubmit, please ensure that you provide the correct grant numbers for the awards you received for your study in the ‘Funding Information’ section

3. Thank you for stating the following financial disclosure:

“China Scholarship Council”

Please state what role the funders took in the study.  If the funders had no role, please state: "The funders had no role in study design, data collection and analysis, decision to publish, or preparation of the manuscript."

If this statement is not correct you must amend it as needed.

Please include this amended Role of Funder statement in your cover letter; we will change the online submission form on your behalf.

4. We noted in your submission details that a portion of your manuscript may have been presented or published elsewhere. “A very small portion of the standing data used in this manuscript was also included in another manuscript currently under review. That study aimed solely to validate our feedback model for estimating feedback control in normal standing. In contrast, the present study investigates how feedback control is adapted across multiple tasks, including normal standing, unipedal standing, step posture, and walking. The two manuscripts use different data-processing methods (including different velocity normalization procedures), address entirely different research questions, and share no overlapping analyses, results, or conclusions.” Please clarify whether this [conference proceeding or publication] was peer-reviewed and formally published. If this work was previously peer-reviewed and published, in the cover letter please provide the reason that this work does not constitute dual publication and should be included in the current manuscript.

5. If the reviewer comments include a recommendation to cite specific previously published works, please review and evaluate these publications to determine whether they are relevant and should be cited. There is no requirement to cite these works unless the editor has indicated otherwise.

[Note: HTML markup is below. Please do not edit.]

Reviewers' comments:

Reviewer's Responses to Questions

**Comments to the Author**

1. Is the manuscript technically sound, and do the data support the conclusions?

Reviewer #1: No

Reviewer #2: Yes

Reviewer #3: No

**********

2. Has the statistical analysis been performed appropriately and rigorously?

Reviewer #1: N/A

Reviewer #2: Yes

Reviewer #3: Yes

**********

3. Have the authors made all data underlying the findings in their manuscript fully available?

The PLOS Data policy

Reviewer #1: Yes

Reviewer #2: Yes

Reviewer #3: Yes

**********

4. Is the manuscript presented in an intelligible fashion and written in standard English?

Reviewer #1: Yes

Reviewer #2: Yes

Reviewer #3: Yes

**********

Reviewer #1: The authors present a study comparing the mechanisms underlying balance during standing and walking, addressing an important question for which substantial gaps in understanding remain. One likely reason for these gaps is that feedback control during walking is inherently difficult to assess, both experimentally and conceptually, due to the closed-loop nature of the system.

While the experiment is carefully conducted and the manuscript is clearly written, I see a major conceptual limitation in the overall study design. Specifically, I do not see how the reported results can be used to answer the stated research question regarding feedback mechanisms. The issue is not one of data quality or analysis, but of methodological suitability.

Major concerns:

The study aims to investigate a feedback (control) mechanism, yet the chosen experimental approach does not allow such a mechanism to be identified. In particular, the approach is ill-posed for inferring feedback dynamics in a closed-loop system, as it does not provide a means to disentangle plant dynamics from feedback control. As a result, the underlying control mechanism cannot be uniquely inferred from the observed behavior.

In this context, it is also important to note that the manuscript makes almost no reference to the substantial body of literature on feedback dynamics in human balance control, primarily in standing but also in walking. This literature explicitly discusses the identifiability limitations of closed-loop balance control and the need for appropriate perturbations or excitation. Several influential studies that are directly relevant to the present work are listed below and should be considered when interpreting feedback in balance control.

More specifically, I see two conceptual problems with the proposed approach.

First, the formulation of the feedback is incorrect. The model equations (eq. 1 in line 182 and eq. 2) do not represent the dynamics of a feedback mechanism. Instead, they describe an open-loop mapping from body kinematics to the force generated by the control mechanism. However, the corrective forces Fcorr also affect PCoM and VCoM, which is not accounted for in the formulation. This self-referential nature—where the sensory input (PCoM,VCoM) affects Fcorr, and Fcorr in turn affects the sensory input—is the core defining feature of feedback control. Ignoring this interdependence breaks the closed-loop description of the system. This mistake leads to biased parameter estimates and, more importantly, to an incorrect representation of the system dynamics.

Second, the experimental approach is limited in its ability to identify feedback (control) mechanisms. Fundamental results from control theory and closed-loop system identification indicate that external perturbations or persistently exciting inputs are generally required to disentangle plant dynamics from feedback control. In the absence of such perturbations, the identification problem becomes ill-conditioned and does not allow unique inference of the underlying control structure. A technical discussion of this issue in the balance control literature is provided by van der Kooij et al. (2005), with similar arguments found in standard system identification textbooks (e.g. Pintelon and Schoukens 2004).

To put this argument in simple terms: sensory systems, neural processing, and the generation of torque all introduce noise, i.e. random fluctuations in the signals of the control system. The properties of this noise (spectral color, magnitude, etc.) are unknown. At the same time, the dynamics of the control system—its structure and parameters—are also unknown. Spontaneous sway behavior during unperturbed stance therefore reflects the combined effect of unknown noise properties and unknown feedback dynamics. Both interact and together give rise to the observed behavior.

When comparing two conditions with different sway patterns, such differences may result from changes in noise properties, changes in feedback dynamics, or a combination of both. In the present experiments, a comparison between walking and standing would require the assumption that internal noise characteristics are identical in both conditions. This assumption is unlikely to hold, as during walking motor noise, and at a minimum also the noise associated with proprioceptive reference to the support surface, differ from those in standing balance. Consequently, it is not possible to determine whether observed differences arise from changes in the control mechanism, changes in noise properties, both, or neither.

The only way to resolve this ambiguity is to apply controlled perturbations and to separate the response to the perturbation from spontaneous sway components. The relationship between perturbation and sway response then characterizes the dynamics of the control system and can be used to identify candidate control mechanisms. This approach is well established in the balance control literature (see Peterka 2002 for a milestone contribution; see also Reimann et al. 2018 and Rafiee & Kiemel 2020 for applications to walking balance).

In summary, feedback control of balance cannot be identified from pure observation when noise sources have unknown characteristics, which constitutes a fundamental limitation when studying closed-loop systems. For the present study, this implies that the proposed experimental approach does not allow the feedback dynamics to be inferred from the collected data.

Peterka, Robert J. “Sensorimotor integration in human postural control.“ Journal of neurophysiology 88, Nr. 3 (2002): 1097–118.

Kooij, Herman van der, Edwin van Asseldonk, und Frans C T van der Helm. “Comparison of different methods to identify and quantify balance control.“ Journal of neuroscience methods 145, Nr. 1–2 (2005): 175–203. https://doi.org/10.1016/j.jneumeth.2005.01.003.

Peterka, Robert J. “Simplifying the complexities of maintaining balance.“ IEEE engineering in medicine and biology magazine : the quarterly magazine of the Engineering in Medicine & Biology Society 22, Nr. 2 (2003): 63–68.

Mergner, T, C Maurer, und R J Peterka. “A multisensory posture control model of human upright stance.“ Progress in brain research 142, Nr. 1 (2003): 189–201. https://doi.org/10.1016/S0079-6123(03)42014-1.

Pasma, J. H., D. Engelhart, A. C. Schouten, H. van der Kooij, A. B. Maier, und C. G.M. Meskers. “Impaired standing balance: The clinical need for closing the loop“. Neuroscience 267 (2014): 157–65. https://doi.org/10.1016/j.neuroscience.2014.02.030.

Maurer, C, und R J Peterka. “A new interpretation of spontaneous sway measures based on a simple model of human postural control.“ Journal of neurophysiology 93, Nr. 1 (2005): 189–200. https://doi.org/10.1152/jn.00221.2004.

Reimann, H., Fettrow, T., Thompson, E. D., & Jeka, J. J. (2018). Neural control of balance during walking. Frontiers in physiology, 9, 1271.

Rafiee, S., & Kiemel, T. (2020). Multiple strategies to correct errors in foot placement and control speed in human walking. Experimental brain research, 238(12), 2947-2963.

Pintelon, Rik, und Johan Schoukens. System identification: a frequency domain approach. John Wiley & Sons, 2012.

Reviewer #2: Summary

Understanding whether common control principles govern balance during standing and walking has implications for motor control theory, clinical assessment, and rehabilitation. This study applies a unified feedback control model to unperturbed walking and three standing postures: bipedal, unipedal, and step posture. The authors estimate position and velocity feedback gains and effective delays from center of mass kinematics and ground reaction forces in 15 young adults. Model fits are generally high across tasks. Feedback delays are longer in walking than standing. Normalized position gains exceed critical stiffness in most conditions. The ratio of position to velocity gains approximates the inverted pendulum eigenfrequency across tasks. The authors interpret this as evidence of shared control principles between standing and walking.

This is a meaningful and well-motivated question. Applying one observable feedback model to both walking and standing without perturbations is novel and potentially valuable. The modeling approach is clearly described in many sections. However, several methodological and interpretive issues limit confidence in the strength of the central claim. These concerns are addressable without new data, but they do require clearer justification, potentially additional analyses, and more careful framing of conclusions.

Overall Assessment

The authors address a meaningful problem in locomotor control. The approach is promising and the results are potentially informative. However, the strength of the “shared principles” conclusion currently exceeds what the analyses may support. Methodological inconsistencies, model assumptions, and interpretation issues complicate direct comparisons across tasks. Clarifying these issues and refining the framing of the conclusions would substantially strengthen the manuscript and improve confidence in its claims.

>> Major Points

1. Inconsistent Delay Identification Across Tasks

Although the manuscript applies a “unified” feedback model, the delay identification procedure differs between standing (R² maximization; lines 189-190) and walking (most negative weighted sum of gains; lines 200-201). This inconsistency limits the comparability of feedback parameters across tasks and challenges the claim of a unified modeling framework (lines 182-185).

This matters because delay directly influences gain estimates. If delay is optimized using different criteria across tasks, then differences in delay and gain magnitude may reflect optimization strategy rather than physiological differences. Since the goal is to argue for shared control principles using a unified framework, then the fitting approach and parameter identification must be comparable or at least shown not to bias the comparison.

To resolve this, I recommend that the authors:

1. Either apply the same delay selection criterion to both tasks and reanalyze the data

2. Or provide a clear(er) rationale for why different optimization criteria are necessary and include a sensitivity analysis showing that the main findings hold under alternative delay selection rules.

Without this, it remains unclear whether the reported task differences reflect control strategy or fitting procedure.

2. Definition and justification of “corrective” forces in walking

The authors remove gravitational components from walking ground reaction forces based on inverted pendulum model assumptions and refers to the remaining signal as “corrective” force (lines 157-160; lines 166-169). My concern is not that this approach is invalid, but that its justification and implications are insufficiently developed for walking (lines 168-169).

Walking, particularly during double support, departs from single inverted pendulum mechanics (lines 168-169). The model's failure to capture walking complexity during double-stance may lead to systematic errors in force decomposition and these errors could propagate to parameter estimates in ways that systematically bias comparisons with standing.

Also, while model fits improved using corrective forces, this improvement does not necessarily validate the decomposition. Because both the preprocessing force decomposition and the feedback model rely on the same pendulum assumptions, there is potential for the preprocessing to shape the data in ways that favor the model fit that sounds circular in the methods (lines 173-175).

I recommend that the authors consider:

> Provide a clearer mathematical description of how corrective forces were computed. Present the equations explicitly and state all assumptions.

> Consider analyzing single stance separately, where pendulum assumptions are more defensible, or justify why inclusion of double stance does not affect conclusions. A sensitivity check might clarify whether decomposition biases parameter estimate.

This issue influences interpretation of walking parameter estimates and therefore the central comparison with standing. More transparent justification would strengthen confidence in the derived walking parameters.

3. Interpretation of the Position-to-velocity Gain Ratio

The authors interpret the finding that the gain ratio approximates √(g/l) as evidence for shared neural control principles (lines 383-388). However, an inverted pendulum like ratio can arise from shared mechanics alone (line 471-472), independent of neural strategy. This is an interesting result but the interpretation should be more cautious.

I recommend that the authors:

> Reframe the interpretation more cautiously. Explicitly distinguish what this finding reveals about neural control versus what it reveals about whole-body mechanics.

> Consider whether deviations from √(g/l) might be more informative than the approximate match. Deviations from √(g/l) across tasks might reveal how the nervous system adapts control parameters to task-specific demands, whereas approximate consistency primarily confirms that both tasks involve stabilizing a pendular body. Task-specific deviations could indicate functional specialization of control strategies.

These changes would acknowledge alternative explanations rather than narrowly interpreting it as direct evidence of shared neural strategy.

4. Influence of Intrinsic Stiffness on Delay and Gain Estimates

The authors acknowledge that intrinsic mechanical stiffness bias feedback parameter estimates, particularly the delay parameter. This is mentioned as a limitation but deserves more serious treatment given its potential to confound all major comparisons.

Task differences alter co-contraction levels and intrinsic stiffness (lines 432-442). Changes in intrinsic stiffness can: shorten delays, alter effective gain estimates, and change system dynamics independently of neural feedback. If intrinsic stiffness varies systematically across tasks, then some reported differences in delay and gain magnitude may reflect mechanical changes rather than changes in feedback control.

I recommend that the authors consider:

> Clarify which conclusions are robust to this confound and which should be interpreted cautiously.

> Consider whether intrinsic stiffness could be explicitly incorporated into a model, or explicitly justify why it is treated as a limitation.

Without this clarification, confidence in interpretating task differences remains limited.

5. Treatment of Phase-Dependent Control in Walking

Walking gains vary across stance phase, yet the authors use mean gains to compare walking with time-invariant standing (lines 204-208; lines 268-272). Averaging simplifies the analysis, but it removes temporal structure, an important and distinguishing feature of walking control.

I recommend that the authors:

> Clarify what is meant by “shared principles” throughout the text. Is it similarity in instantaneous control structure, average gain magnitude, or structural relationships such as the gain ratio?

> Address whether phase-dependent control in walking represents a fundamentally different strategy or a modulation of the same underlying control strategy.

This point would strengthen conceptual clarity.

6. Inverted Pendulum Assumptions and Multisegmented Contributions

The discussion relies heavily on single inverted pendulum interpretations, including critical stiffness and eigenfrequency arguments. While the inverted pendulum model is useful and widely used, both standing and walking involve multi-link coordination. My concern is not that the inverted pendulum model is inappropriate, but that conclusions may extend beyond what the model can justify

I recommend that the authors consider:

> Explaining the specific deviations (multi-link coordination, ankle vs. hip strategies) and their implications for the model. Relate findings to literature evaluating when single pendulum models are adequate and when multi-link behavior dominates.

> Clarifying how much of the observed behavior can reasonably be attributed to inverted pendulum mechanics versus multi‑segment control.

This will prevent overgeneralization and strengthen the credibility of the conclusions.

7. Task Definitions: Unipedal and Step Posture Clarifications

Unipedal Stance (lines 129-130): The position of the non-stance leg is not clearly described. This detail matters because leg position alters stability demands.

Please specify:

> Was the leg held behind (like initial swing/toe-off), ahead (like terminal swing/pre-heel-strike), or adjacent (like mid-swing)?

> Were hip and knee angles standardized?

> Was this controlled across participants and trials?

The introduction states these postures resemble behaviors during single support in walking. If that is the intent, specify which swing phase posture was modeled and justify the choice.

Step Posture (lines 132-135): The step posture imposed equal weight distribution between feet. In natural double support, weight distribution is dynamic and asymmetric.

Please justify:

> Why 50 percent weight distribution was selected.

> Why initial or terminal double support, which may better resemble walking mechanics, was not modeled.

> Whether a single inverted pendulum model is appropriate for bipedal stance with two contact points and a base of support.

Clarification of whether the step posture is meant to represent double‑support mechanics or simply serve as an additional standing configuration needs to be stated clearly.

>>Minor Points

Introduction

> The first two paragraphs are largely under-cited. Add references on feedback control in standing and walking, and distinct feedback strategies.

> Improve organization and conceptual flow. The jump from standing vs. walking differences to feedback models to XCoM concept could be smoother. Consider reorganizing.

> Introduce and explain XCoM more clearly when first mentioned (lines 69-70). The explanation of XCoM and its predictions comes several paragraphs after it is first mentioned (lines 88-90).

Methods

> Use SI units consistently. Report walking speed as 1.25 m/s and optionally include km/h in parentheses (line 125).

> Justify exclusion of the first 6 seconds and last 10 seconds from standing trials (line 161).

> Specify how ground reaction forces collected during standing postures. Confirm whether participants stood on the instrumented treadmill used during the walking trials (lines 128-141).

Results

> Table 1 reports partial eta squared values but does not interpret them. Provide brief interpretation of effect sizes and discuss practical significance.

> State what level of explained variance (R2) is considered acceptable for drawing conclusions (lines 248-249).

Discussion

> Clarify what is novel if similarities primarily reflect shared whole-body mechanics. Clarify what the study reveals about neural control versus body mechanics.

How do the authors reconcile finding both similarities (gain ratios) and large differences (delays, individual gain magnitudes)? What level of difference would falsify the 'shared principles' hypothesis?

The authors should consider reframing the title to reflect both commonalities and task-specific differences to better match the actual findings

The authors should be more explicit about what constitutes a "shared principle" vs. task-specific adaptation. Is it just the gain ratio, or are there other features?

>> Recommendation: Minor Revision

The authors address an important problem and use a promising modeling approach. However, several methodological inconsistencies and interpretive issues limit confidence in the central “shared principles” conclusion. These concerns appear addressable through clearer justification, targeted sensitivity analyses, and more careful framing. With these revisions, the manuscript could make a meaningful contribution to understanding feedback control in standing and walking.

Reviewer #3: General comments

This paper addresses an interesting open question in motor control, namely, the adaptability of control schemes between different motor tasks. This paper aims to examine how the parameters in a common feedback control structure compare for standing and walking.

Unfortunately, conceptual flaws in the paper make the argument presented entirely unconvincing. To plausibly demonstrate that a feedback control system is playing a role in a system requires at least two things: a target or set point of some quantity of relevance to the system’s performance, and a demonstration that some form of state feedback is able to drive the system to that target value. The “feedback models” in the authors’ Eqs. (1) and (2) do neither.

This paper considers a point mass walker with its state given by the position and velocity of the center of mass (CoM). What the authors have demonstrated is that, for both standing and walking, the state is linearly correlated to a reasonable degree with the forces generated at the next time. They also identify the time lag that yields the best correlation. But they never show that this supposed control is being used to regulate the actual walking dynamics in any way: for that, one would presumably have to show how the given force is used to create the next CoM state. Indeed, the paper refers to “corrective ground reaction forces” multiple times without ever stating what, exactly, is being corrected. It is merely assumed that the forces they have measured are being used to correct something, but what is not at all clear.

They also find that the correlation parameters obtained in the four cases examined differ and thereby interpret this as “adaptation”. This is at best an hypothesis, and does not indicate a common controller is active in the different situations. The fact that one can find a reasonable linear correlation between the CoM state and force for the different tasks hardly shows that they are using the same controller architecture. Correlation is not causation, but even assuming the CoM state is causing the subsequent force, any mechanism for doing that would cause the two cases to show correlations and, assuming the controller was differentiable, they would both have a linear limit and, hence, could be expected to reasonably satisfy Eqs. (1) or (2). So the fact that one can estimate parameters in all of the cases in no way convincingly demonstrates “adaptation”.

The data collection and analysis methodologies are sound and, as such, the paper has potential to be published in PLOS ONE. However, the interpretations are poorly drawn because of the above-indicated fundamental problems. I cannot, therefore, recommend the paper for publication in its current form. In addition to addressing the specific comments below, the authors need to rewrite the introduction and discussion to more convincingly relate the simple correlation analyses they are doing to the claims about control they are making.

Specific comments

1. The authors cite only one model of feedback control in walking (the recent paper by Afshcrift et al.). There are many such models. A more representative list and a brief discussion how this study’s definition of control fits in with them will greatly help the manuscript and may help justify your claims about control and adaptation.

2. Eq. (1) is a standard AR model with fixed coefficients, whereas Eq. (2) has time (or state) dependent coefficients that are estimated at each phase of a step. The second model is therefore not just an adaptation of the first (or vice versa). To show adaptation, you would need to have exactly the same model in both cases, and show that only its parameters needed to change to obtain comparable results.

3. Furthermore, the data you’re fitting in each case is not in the same category: standing gives data that is close to Brownian motion, walking is more like a noisy periodic signal. The time scales over which things are happening in both cases are not remotely the same, so the idea that the same controller is just “adapting” from one case to the next is doubtful.

4. My understanding is that for standing you used 46 seconds of 50 Hz sampled data for the regression, so that’s 2300 data points. Presumably this data appears to be similar to a random walk (in the sense of a random process). In contrast, for walking, you used 5 minutes of walking data, so 300 seconds (and about 300 steps) which, sampled at 50Hz gives 15,000 data points. But you estimate each gain at each of 100 points in a step, so only 300 points are used for each such estimate. This is not only confusing, in the end these regression calculations are not similar to each other so it’s difficult to see how you can compare the two from that point of view. And, again, just because you manage to find reasonably good linear regressions for the different tasks does not imply that the same underlying controller is adapting from one to the other.

5. Finally, as you point out, standing and walking are quite different in that the latter is rhythmic. Hence, the dynamics observed in each case come from very different physiological processes (e.g. walking involves a CPG, standing does not). So, again, it seems highly doubtful that such different tasks use the same underlying control scheme that is merely “adapted” from one to the other.

6. Your study of control only looks at force production. But, as the authors know and have discussed in other works, basic stepping properties (e.g. step width) are known to be very important to maintaining stable walking. Indeed, the very point of Hof’s XCoM and MoS concepts is to look at the relationship between the CoM and the foot position (or, more precisely, the center of pressure location). Why is it that you don’t need to pay any attention to where the feet are placed in this study of control?

7. It would be good to show representative data. Since you estimated K, B and tau for each phase of walking, it would be good to show what those time series looked like when plotted against phase: are they just noisy, or are they structured in some way?

**********

what does this mean?). If published, this will include your full peer review and any attached files.

If you choose “no”, your identity will remain anonymous but your review may still be made public.

**Do you want your identity to be public for this peer review?** For information about this choice, including consent withdrawal, please see our Privacy Policy

Reviewer #1: **Yes:**  Lorenz Assländer

Reviewer #2: No

Reviewer #3: No

**********

[NOTE: If reviewer comments were submitted as an attachment file, they will be attached to this email and accessible via the submission site. Please log into your account, locate the manuscript record, and check for the action link "View Attachments". If this link does not appear, there are no attachment files.]

To ensure your figures meet our technical requirements, please review our figure guidelines: https://journals.plos.org/plosone/s/figures

You may also use PLOS’s free figure tool, NAAS, to help you prepare publication quality figures: https://journals.plos.org/plosone/s/figures#loc-tools-for-figure-preparation.

NAAS will assess whether your figures meet our technical requirements by comparing each figure against our figure specifications.

# Author comment: Author response to Decision Letter 1

**DOI:** 10.1371/journal.pone.0355849.r002

29 Apr 2026

We thank you and the reviewers for the time and effort invested in reviewing our manuscript and for providing many valuable comments and suggestions. We have carefully revised the manuscript in response to the feedback. In particular, based on the reviewers’ comments, we now refer to our model as a stabilization model rather than a feedback model, have rephrased the introduction, and have revised the description of the aim of the study from investigating feedback control to focusing on the overall stabilizing mechanisms. A detailed point-by-point response to the reviewers’ comments is provided in the file named “Response to reviewers,” and we have also uploaded both a clean revised manuscript and a version with changes highlighted.

Attachment Submitted filename: Response to reviewers.docx

# Peer review: Decision Letter 1

**DOI:** 10.1371/journal.pone.0355849.r003

**Authors:** Anne Martin

8 Jun 2026

Dear Dr. Bruijn,

Thank you for submitting your manuscript to PLOS ONE. After careful consideration, we feel that it has merit but does not fully meet PLOS ONE’s publication criteria as it currently stands. Therefore, we invite you to submit a revised version of the manuscript that addresses the points raised during the review process.

Please submit your revised manuscript by Jul 23 2026 11:59PM. If you will need more time than this to complete your revisions, please reply to this message or contact the journal office at plosone@plos.org. When you're ready to submit your revision, log on to https://www.editorialmanager.com/pone/ and select the 'Submissions Needing Revision' folder to locate your manuscript file.

- A letter that responds to each point raised by the academic editor and reviewer(s). You should upload this letter as a separate file labeled 'Response to Reviewers'.
- A marked-up copy of your manuscript that highlights changes made to the original version. You should upload this as a separate file labeled 'Revised Manuscript with Track Changes'.
- An unmarked version of your revised paper without tracked changes. You should upload this as a separate file labeled 'Manuscript'.

If you would like to make changes to your financial disclosure, please include your updated statement in your cover letter. Guidelines for resubmitting your figure files are available below the reviewer comments at the end of this letter.

If applicable, we recommend that you deposit your laboratory protocols in protocols.io to enhance the reproducibility of your results. Protocols.io assigns your protocol its own identifier (DOI) so that it can be cited independently in the future. For instructions see: https://journals.plos.org/plosone/s/submission-guidelines#loc-laboratory-protocols. Additionally, PLOS ONE offers an option for publishing peer-reviewed Lab Protocol articles, which describe protocols hosted on protocols.io. Read more information on sharing protocols at https://plos.org/protocols?utm_medium=editorial-email&utm_source=authorletters&utm_campaign=protocols.

As the corresponding author, your ORCID iD is verified in the submission system and will appear in the published article. PLOS supports the use of ORCID, and we encourage all coauthors to register for an ORCID iD and use it as well. Please encourage your coauthors to verify their ORCID iD within the submission system before final acceptance, as unverified ORCID iDs will not appear in the published article. *Only* the individual author can complete the verification step; PLOS staff *cannot* verify ORCID iDs on behalf of authors.

We look forward to receiving your revised manuscript.

Kind regards,

Anne E. Martin

Academic Editor

PLOS One

Journal Requirements:

1. If the reviewer comments include a recommendation to cite specific previously published works, please review and evaluate these publications to determine whether they are relevant and should be cited. There is no requirement to cite these works unless the editor has indicated otherwise.

Additional Editor Comments:

The reviewer raises important points about if the fits are consistent and robust. Please add in additional tests to demonstrate that they are.

[Note: HTML markup is below. Please do not edit.]

Reviewer's Responses to Questions

**Comments to the Author**

Reviewer #1: (No Response)

**********

2. Is the manuscript technically sound, and do the data support the conclusions?

Reviewer #1: Partly

**********

3. Has the statistical analysis been performed appropriately and rigorously?

Reviewer #1: Yes

**********

4. Have the authors made all data underlying the findings in their manuscript fully available?

The PLOS Data policy

Reviewer #1: Yes

**********

5. Is the manuscript presented in an intelligible fashion and written in standard English?

Reviewer #1: Yes

**********

Reviewer #1: I thank the authors for addressing my previous comments. The revised manuscript has improved in clarity, particularly regarding the interpretation of the model. I appreciate that the authors now explicitly acknowledge that the experimental design does not allow identification of feedback control dynamics in a closed-loop system, and that the estimated parameters should not be interpreted as feedback gains or delays.

However, clarifying interpretation does not resolve the more fundamental issue of whether the parameters are identifiable and can be reliably estimated from the available data. In closed-loop systems driven by spontaneous sway without external excitation, different parameter combinations can lead to near-equivalent fits. This is further amplified as estimates may be dominated by noise structure rather than underlying system properties.

At present, the manuscript does not provide evidence that the proposed parameterization yields stable and uniquely identifiable estimates under these conditions. This is critical because even if the parameters are interpreted only as “lumped stabilizing mechanisms,” such interpretations are only meaningful if the mapping from data to parameters is sufficiently robust.

To test and demonstrate identifiability and estimator stability showing that the proposed approach does work, I suggest the following minimal set of analyses:

(i) The repeatability of the estimated parameters across the 5 measured trials should be quantified and reported. A straightforward approach is to compute intra-class correlation coefficients (or related measures of between-trial variability) across the repeated measurements. This would provide evidence regarding the stability and reliability of the parameter estimates under identical experimental conditions.

(ii) A second important test would be to estimate parameters while fixing the delay at different values to assess whether the delay is identifiable independently of gain parameters. This is important, as there is a risk that the changes in delay values are absorbed into changes in gain parameters.

Given the absence of external perturbations or other sources of persistent excitation, such analyses are required to assess whether the reported parameters reflect robust features of the system or are artifacts of an under-excited experimental approach and potentially poorly constrained estimation problem. For general methodological guidance of modelling approaches, I would like to highlight the following reference.

Wilson, Robert C., und Anne Ge Collins. “Ten Simple Rules for the Computational Modeling of Behavioral Data“. eLife 8 (November 2019): e49547. https://doi.org/10.7554/eLife.49547.

**********

what does this mean?). If published, this will include your full peer review and any attached files.

If you choose “no”, your identity will remain anonymous but your review may still be made public.

**Do you want your identity to be public for this peer review?** For information about this choice, including consent withdrawal, please see our Privacy Policy

Reviewer #1: **Yes:**  Lorenz Assländer

**********

[NOTE: If reviewer comments were submitted as an attachment file, they will be attached to this email and accessible via the submission site. Please log into your account, locate the manuscript record, and check for the action link "View Attachments". If this link does not appear, there are no attachment files.]

To ensure your figures meet our technical requirements, please review our figure guidelines: https://journals.plos.org/plosone/s/figures

You may also use PLOS’s free figure tool, NAAS, to help you prepare publication quality figures: https://journals.plos.org/plosone/s/figures#loc-tools-for-figure-preparation.

NAAS will assess whether your figures meet our technical requirements by comparing each figure against our figure specifications.

# Author comment: Author response to Decision Letter 2

**DOI:** 10.1371/journal.pone.0355849.r004

1 Jul 2026

We would like to thank you and the reviewer for the time and effort invested in reviewing our revised manuscript and for providing comments and suggestions. We have carefully considered the reviewer’s key concerns regarding the reliability and identifiability of estimated parameters, and have conducted additional analyses to address these questions. We have also added an appendix to provide the detailed description of these analyses and the corresponding results. A detailed point-by-point response to the reviewers’ comments is provided in the file named “Response to Reviewers,” and we have also uploaded a revised manuscript with the changes highlighted.

Attachment Submitted filename: Response_to_Reviewers_auresp_2.docx

# Peer review: Decision Letter 2

**DOI:** 10.1371/journal.pone.0355849.r005

**Authors:** Anne Martin

9 Aug 2026

PONE-D-25-66922R2

Different stabilizing mechanisms but a common task-level aim in standing and walking

PLOS One

Dear Dr. Bruijn,

In light of the information you have provided, we have rescinded the current acceptance decision and, on behalf of the Academic Editor, are issuing a Revision decision. This will allow you to submit a corrected version of the manuscript.

Please submit your revised manuscript by Oct 05 2026 11:59PM. If you will need more time than this to complete your revisions, please reply to this message or contact the journal office at plosone@plos.org. When you're ready to submit your revision, log on to https://www.editorialmanager.com/pone/ and select the 'Submissions Needing Revision' folder to locate your manuscript file.

Please include the following items when submitting your revised manuscript:

- Provide a detailed Response to the Editor explaining the analytical error, the corrections made, and the extent to which the results and conclusions have changed.
- A marked-up copy of your manuscript that highlights changes made to the original version. You should upload this as a separate file labeled 'Revised Manuscript with Track Changes'.
- An unmarked version of your revised paper without tracked changes. You should upload this as a separate file labeled 'Manuscript'.

If you would like to make changes to your financial disclosure, please include your updated statement in your cover letter. Guidelines for resubmitting your figure files are available below the reviewer comments at the end of this letter.

If applicable, we recommend that you deposit your laboratory protocols in protocols.io to enhance the reproducibility of your results. Protocols.io assigns your protocol its own identifier (DOI) so that it can be cited independently in the future. For instructions see: https://journals.plos.org/plosone/s/submission-guidelines#loc-laboratory-protocols. Additionally, PLOS ONE offers an option for publishing peer-reviewed Lab Protocol articles, which describe protocols hosted on protocols.io. Read more information on sharing protocols at https://plos.org/protocols?utm_medium=editorial-email&utm_source=authorletters&utm_campaign=protocols.

As the corresponding author, your ORCID iD is verified in the submission system and will appear in the published article. PLOS supports the use of ORCID, and we encourage all coauthors to register for an ORCID iD and use it as well. Please encourage your coauthors to verify their ORCID iD within the submission system before final acceptance, as unverified ORCID iDs will not appear in the published article. Only the individual author can complete the verification step; PLOS staff cannot verify ORCID iDs on behalf of authors.

We look forward to receiving your revised manuscript.

Kind regards,

Andreas Paris, PhD

Associate Editor

PLOS One

Journal Requirements:

If the reviewer comments include a recommendation to cite specific previously published works, please review and evaluate these publications to determine whether they are relevant and should be cited. There is no requirement to cite these works unless the editor has indicated otherwise.

[Note: HTML markup is below. Please do not edit.]

Reviewers' comments:

Reviewer's Responses to Questions

**Comments to the Author**

Reviewer #1: All comments have been addressed

**********

2. Is the manuscript technically sound, and do the data support the conclusions?

Reviewer #1: Yes

**********

3. Has the statistical analysis been performed appropriately and rigorously?

Reviewer #1: Yes

**********

4. Have the authors made all data underlying the findings in their manuscript fully available?

The PLOS Data policy

Reviewer #1: Yes

**********

5. Is the manuscript presented in an intelligible fashion and written in standard English?

Reviewer #1: Yes

**********

Reviewer #1: I would like to thank the authors for addressing my concerns in such a substantial manner. I do see my concerns partly reflected in the results, as the SEM Values are partly very large. However, the clear presentation of the reliability and based on the more reliable estimates of e.g. the stiffness, the interpretations seem sound and I do not have any further comments.

**********

what does this mean?). If published, this will include your full peer review and any attached files.

If you choose “no”, your identity will remain anonymous but your review may still be made public.

**Do you want your identity to be public for this peer review?** For information about this choice, including consent withdrawal, please see our Privacy Policy

Reviewer #1: **Yes:**  Lorenz Assländer

**********

[NOTE: If reviewer comments were submitted as an attachment file, they will be attached to this email and accessible via the submission site. Please log into your account, locate the manuscript record, and check for the action link "View Attachments". If this link does not appear, there are no attachment files.]

To ensure your figures meet our technical requirements, please review our figure guidelines: https://journals.plos.org/plosone/s/figures

You may also use PLOS’s free figure tool, NAAS, to help you prepare publication quality figures: https://journals.plos.org/plosone/s/figures#loc-tools-for-figure-preparation.

NAAS will assess whether your figures meet our technical requirements by comparing each figure against our figure specifications.

# Author comment: Author response to Decision Letter 3

**DOI:** 10.1371/journal.pone.0355849.r006

7 Sep 2026

We thank you and the reviewer for the time and effort invested in reviewing previous versions of the manuscript. After acceptance of the paper, we identified an error in the code that had affected some of the results on walking. We therefore reran the code and recalculated the relevant results. Fortunately, the overall conclusion remained largely unchanged. We have revised the corresponding descriptions throughout the manuscript and updated the code, figures and tables accordingly. The specific changes and explanation can be found in file “Response to the Editor and reviewers”. The changes are incorporated in a clean revised manuscript and highlighted in a marked version.

Attachment Submitted filename: Response to the Editor and reviewers.docx

# Peer review: Decision Letter 3

**DOI:** 10.1371/journal.pone.0355849.r007

**Authors:** Anne Martin

25 Sep 2026

Different stabilizing mechanisms but a common task-level aim in standing and walking

PONE-D-25-66922R3

Dear Dr. Bruijn,

We’re pleased to inform you that your manuscript has been judged scientifically suitable for publication and will be formally accepted for publication once it meets all outstanding technical requirements.

Within one week, you’ll receive an e-mail detailing the required amendments. When these have been addressed, you’ll receive a formal acceptance letter and your manuscript will be scheduled for publication.

An invoice will be generated when your article is formally accepted. Please note, if your institution has a publishing partnership with PLOS and your article meets the relevant criteria, all or part of your publication costs will be covered. Please make sure your user information is up-to-date by logging into Editorial Manager at Editorial Manager® and clicking the ‘Update My Information' link at the top of the page. For questions related to billing, please contact billing support.

If your institution or institutions have a press office, please notify them about your upcoming paper to help maximize its impact. If they’ll be preparing press materials, please inform our press team as soon as possible -- no later than 48 hours after receiving the formal acceptance. Your manuscript will remain under strict press embargo until 2 pm Eastern Time on the date of publication. For more information, please contact onepress@plos.org.

Kind regards,

Anne E. Martin

Academic Editor

PLOS One

Additional Editor Comments (optional):

Reviewers' comments:

Reviewer's Responses to Questions

**Comments to the Author**

Reviewer #1: All comments have been addressed

**********

2. Is the manuscript technically sound, and do the data support the conclusions?

Reviewer #1: Yes

**********

3. Has the statistical analysis been performed appropriately and rigorously?

Reviewer #1: Yes

**********

4. Have the authors made all data underlying the findings in their manuscript fully available?

The PLOS Data policy

Reviewer #1: Yes

**********

5. Is the manuscript presented in an intelligible fashion and written in standard English?

Reviewer #1: Yes

**********

Reviewer #1: The corrected results look sound to me and the reason for the correction is well explained. I have no further comments.

**********

what does this mean?). If published, this will include your full peer review and any attached files.

If you choose “no”, your identity will remain anonymous but your review may still be made public.

**Do you want your identity to be public for this peer review?** For information about this choice, including consent withdrawal, please see our Privacy Policy

Reviewer #1: No

**********

# Editor report: Acceptance letter

**DOI:** 10.1371/journal.pone.0355849.r008

**Authors:** Anne Martin

PONE-D-25-66922R3

PLOS One

Dear Dr. Bruijn,

I'm pleased to inform you that your manuscript has been deemed suitable for publication in PLOS One. Congratulations! Your manuscript is now being handed over to our production team.

At this stage, our production department will prepare your paper for publication. This includes ensuring the following:

* All references, tables, and figures are properly cited

* All relevant supporting information is included in the manuscript submission,

* There are no issues that prevent the paper from being properly typeset

You will receive further instructions from the production team, including instructions on how to review your proof when it is ready. Please keep in mind that we are working through a large volume of accepted articles, so please give us a few days to review your paper and let you know the next and final steps.

Lastly, if your institution or institutions have a press office, please let them know about your upcoming paper now to help maximize its impact. If they'll be preparing press materials, please inform our press team within the next 48 hours. Your manuscript will remain under strict press embargo until 2 pm Eastern Time on the date of publication. For more information, please contact onepress@plos.org.

You will receive an invoice from PLOS for your publication fee after your manuscript has reached the completed accept phase. If you receive an email requesting payment before acceptance or for any other service, this may be a phishing scheme. Learn how to identify phishing emails and protect your accounts at https://explore.plos.org/phishing.

If we can help with anything else, please email us at customercare@plos.org.

Thank you for submitting your work to PLOS One and supporting open access.

Kind regards,

PLOS One Editorial Office Staff

on behalf of

Dr. Anne E. Martin

Academic Editor

PLOS One
